# Through-Mask Electrochemical Micromachining with Reciprocating Foamed Cathode

**DOI:** 10.3390/mi11020188

**Published:** 2020-02-11

**Authors:** Chenhao Zhao, Pingmei Ming, Xinmin Zhang, Ge Qin, Jiwen Shen, Liang Yan, Xingshuai Zheng, Jun Cao

**Affiliations:** Institute of Non-traditional Machining & Equipment, Henan Polytechnic University, Jiaozuo 454000, China; zhaochenhao930@163.com (C.Z.); qinge@hpu.edu.cn (G.Q.); yanliang@hpu.edu.cn (L.Y.); xshuai_zheng@163.com (X.Z.); cavan@hpu.edu.cn (J.C.)

**Keywords:** through-mask electrochemical machining, foamed cathode, electrochemical machining, magnetic field effects, reciprocating movement

## Abstract

A through-mask electrochemical micromachining process with a foamed cathode (foamed-cathode through-mask electrochemical micromachining (TMEMM)) has recently been proposed involving micro-scale surface microstructures with a high geometric consistency that are fabricated on the curved-surface workpiece. In this paper, to make the foamed-cathode TMEMM process more cost-efficient in the applications, significant modifications are made to this process and an upgraded version of the foamed-cathode TMEMM process is developed. In this modified process, the sandwich-like unit (including the foamed cathode, mask, and workpiece) is closely assembled by the magnetic field force instead of the conventionally-used mechanical force and is kept moving up-and-down inside the electrolyte, avoiding the use of the traditional pump-driven circulation for the electrode process. Experiments are carried out to evaluate the machining effect of this modified TMEMM for fabricating micro-dimples. The research results verify that this modified TMEMM process can produce highly uniform micro-dimples whose minimum CV (coefficient of variation) values in depth and in diameter are 5.4% and 1.9%, respectively, with smooth surfaces of the minimum Ra being 0.21–0.35 µm. These values are smaller than those previously reported. This results in the positive effects on the mass transfer driven by magnetohydrodynamic convection induced by the magnetic field within the interelectrode and the foamed electrode.

## 1. Introduction

For decades, engineers and scientists have been deliberately designing and manufacturing a variety of surface microstructures to improve the properties and functions of products. These properties and functions typically include the bio-compatibility of medical implants [1], resistance to friction and abrasion [2], and extreme wettability [3], etc. To form surface microstructures, various techniques have been developed, for example, chemical etching [4], ultrasonic vibration machining [5], abrasive jet machining [6], laser beam machining [7], and through mask electrochemical micromachining (TMEMM) [8]. Among them, TMEMM, based on the anodic dissolution principle, has a lot of advantages over other techniques, such as no formation of residual stress and recast layer on the processed surface and no limitation in the mechanical properties of the workpiece material [9,10,11,12]. It is popular in shaping surface microstructures. In the TMEMM process, the patterned through-mask is covered on the workpiece, and the unmasked areas are selectively dissolved to generate recessed microstructures. In a standard TMEMM, the through-mask is directly patterned on the workpiece by the photolithographic process and cannot be removed for repeated reuse; hence, traditional TMEMM is very tedious and expensive. In addition, the photoresist through-mask cannot be well formed on the curved surface; as a result, the traditional TMEMM process is somewhat limited in industrial applications. Consequently, effort has been made to facilitate the preparation of the through-mask by researchers. For instance, Landolt et al. and Chauvy et al. [13,14] employed patterned oxide film as the through-mask to texturize titanium. Asoh et al. [15] presented a novel TMEMM process to accomplish the formation of large-scale microstructures just by using the self-assembled polystyrene colloidal crystal particles as the through-mask. In an unusual way, Schönenberger et al. [16] and Costa et al. [17,18] bonded the through-mask to the cathode surface instead of the workpiece surface (anode) to carry out the micro-pattern transferring operation. Recently, Qu et al. [19,20,21] further optimized the preparation of the through-mask of this kind of cathode-typed TMEMM by using the removable dry film through-mask, resulting in high cost-effectiveness.

Unlike the conventional TMEMM processes using the bonded through-mask, a removable and reusable through-mask electrochemical machining process was exploited by Zhu et al. [22,23,24]. In this novel process, the mask is pressed mechanically against the workpiece, and thus, it can be reused repeatedly, showing a favorable operational convenience and cost-effective superiority. Additionally, in this novel process, Qu et al. [25,26,27,28] used polydimethylsiloxane (PDMS) film as the reusable mask due to its good pliancy property. However, fixture of the reusable through-mask on the workpiece is still a significant issue. Normally, the reusable flexible film-typed mask cannot be fixed readily by the traditional mechanical measures because a well-defined interelectrode gap (also as the mass-transfer passage) is required, and thus, the machining accuracy of the reusable through-mask electrochemical micromachining (EMM) is not very satisfactory. 

With further effort to improve the reusable through-mask EMM, our research group modified it by filling pliant porous insulation film with good seepage capacity into the gap between the cathode and the through-mask [29]. This modified EMM is termed the gap-filled through-mask EMM. It was found that textured microstructures can be evenly shaped both on the planar and cylindrical workpieces due to the good adherence of the through-mask to the workpiece with the aid of the porous film. Unfortunately, the material removal rate of this gap-filled TMEMM is relatively low because of the limited mass transfer rate within the interconnected pores of the filled film.

**Figure 1:**
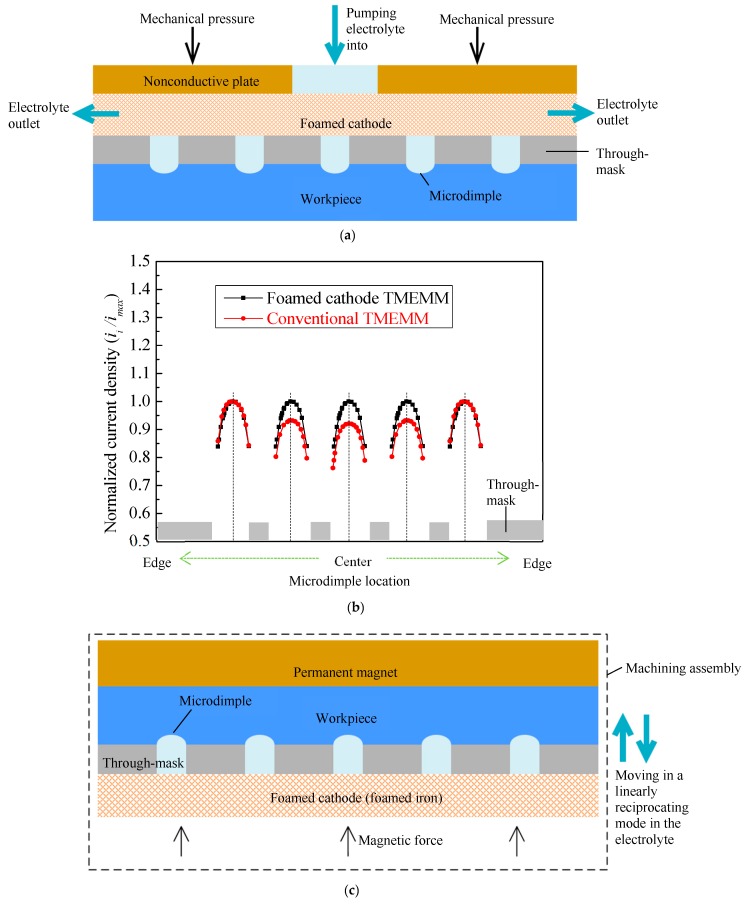
Schematic diagram of the foamed cathode through mask electrochemical micromachining (TMEMM) process with different through-mask fixture manner and mass transfer methods. (**a**) Pressed by the mechanical force, driven by the pump; (**b**) theoretical current distribution within the machined area and across the entire workpiece; (**c**) pressed by the magnetic force, driven by mechanical motion.

Therefore, up to now, almost all TMEMM processes have not satisfied the engineering applications very well. The dimensional uniformity or geometric consistency of TMEMMed surface microstructures is less desirable. In order to solve this issue, our research group further proposed a foamed cathode TMEMM process. In the foamed cathode TMEMM, a foamed metal film with a porosity of above 95% is introduced as both the cathode and the mass transfer media, and it directly contacts the reusable through-mask under an appropriate external pressure; thus, the foamed cathode, the through-mask, and the anode form a sandwich-typed assembly, as shown in Figure 1a. Our research [30] shows that the foamed cathode TMEMM is able to achieve better geometric consistency in the fabricated microstructures mainly due to the significantly uniform current distribution on the entire machining areas (as shown in Figure 1b) and the excellent mass transfer conditions within the interelectrode gap.

This paper focuses on investigating the modification version of the foamed cathode TMEMM process which is enabled with optimized mass transfer conditions and facilitative fixture of the through-mask. Traditionally, as shown in Figure 1a, the mass transfer within the interelectrode gap during TMEMM is driven by a pump, and the outlet and inlet for the electrolyte circulation system are generally fixed, which means the flow path and flow velocity distribution inside the interelectrode gap are relatively changeless. Accordingly, uneven distribution of the flow field within the interelectrode gap in the traditional pump-driven TMEMM is inevitable, and this situation would be more serious when the machining area is very large. On the other hand, magnetic field forces are sometimes very functional to flexibly fix the metal film, and they are also the source that induces magnetohydrodynamic (MHD) effects [31], which are frequently used to create a natural convection for electrochemical reaction systems. In fact, magnetic field assisted electrochemical machining including anodic dissolution and electrodeposition has been widely studied and employed. Some summaries about the fundamental aspects of MHD effects on electrochemical reactions and processes from earlier research activities were well reported by Fahidy et al. [32,33,34]. Pa [35] improved the surface finish of the machined microstructures and machining efficiency by superimposing the magnetic field on the electrochemical micromachining process. Fan et al. [36] reported that the stray-current corrosion was reduced, and thus, the machining accuracy was enhanced if the magnetic field was properly superimposed during electrochemical machining. Bund et al. [37] investigated the influence of an external magnetic field on the electrochemical behavior of Cu and Ni electrodeposition.

In this paper, we evaluate the real effect of using the modified foamed cathode TMEMM process by fabricating a micro-dimple. In the modified foamed cathode TMEMM, the reusable through-mask is pressed against the workpiece by the magnetic field force rather than the mechanical force, and the sandwich-like assembly including the foamed cathode, through-mask, and anode is moved quickly in a linearly reciprocating way to self-circulate the electrolyte within the assembly without the aid of the pump. In this case, the anodic dissolution takes place under the cooperation of MHD-driven natural convection and mechanical-driven forced convection as well as magnetic field induced effects. In the following sections, the machining principle and experimental verification are introduced, and the effects of the key process factors on the machining characteristics are evaluated experimentally.

## 2. Principle of the Foamed Cathode TMEMM under the Magnetic Field

Figure 1c shows the schematic view of the modified foamed cathode TMEMM. The through-mask was placed between the stainless steel sheet workpiece and the foamed iron film, directly contacting each other and hence forming a sandwich-like machining assembly. A permanent magnet was fixed on the workpiece to provide a magnetic field force to hold the foamed cathode, and thereby, the reusable through-mask film between the foamed cathode and the workpiece was tightly and uniformly pressed against the workpiece. The machining assembly was driven to move up and down in a linearly reciprocating mode inside the electrolyte, which was initially static without agitation. In such case, the electrolyte was forced to flow into the cathode’s interconnected pores and then to flow away the assembly, realizing self-circulation of the electrolyte within the interelectrode gap. Meanwhile, the anodic dissolution took place under the magnetic field, which can induce vigorous natural convection in the electrochemical reaction system [38]. Under such circumstances, the mass transfer for the anodic process was controlled cooperatively by the forced convection and the magnetic field-driven natural convection. Therefore, for this proposed TMEMM process, besides convenient fixation of the removable through-mask on the workpiece, more uniform mass transfer field was expected, even without using a circulation driving pump.

## 3. Experimentation

**Figure 2:**
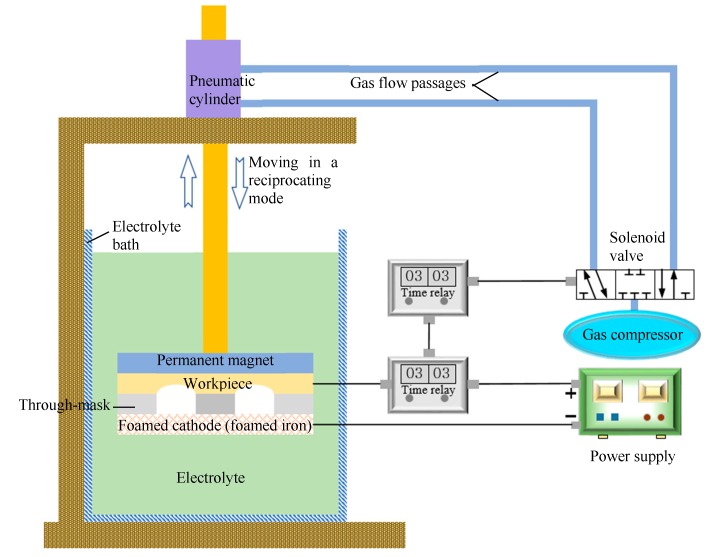
Schematic view of the experimental system for the modified TMEMM process.

The experimental system used for this study, shown in Figure 2, consisted mainly of the machining assembly, driving unit for the reciprocation movement, electrolyte bath, and power supply. The driving unit was a pneumatic cylinder system. Moving speed and reciprocation distance during reciprocation movement can be regulated.

A 1.5 mm thick foamed iron film with a porosity of about 95% and an interconnect micropore size of about 0.1–0.2 mm was used as the cathode. A stainless steel (SUS304) plate with a diameter of 40 mm served as the workpiece. A NdFeB permanent magnet with the surface magnetic flux density of 0.2 T was placed on the back of the workpiece. The single-travel distance of the machining assembly was 0.15 m, and the single-travel time was set to 0.3, 0.5, and 0.7 s, corresponding to average moving speeds of 0.5, 0.3, and 0.2 m/s, respectively. The through-mask (polyvinyl chloride, PVC) containing 121 regularly distributed Φ 0.2 mm perforations with a 1 mm pitch was prepared by the CNC drilling process. A 10 wt% NaNO_3_ solution was used as the electrolyte. Voltages ranging from 6.5 to 9 V were applied to the anodic dissolution. The machining time was 5 min. The process conditions are listed in Table 1. Two machining modes including the moving downwards single-travel mode and moving upwards and downwards double-travel mode were examined experimentally. In the moving downwards single-travel mode, the anodic process took place only when the machining assembly moved downwards, while in the moving upwards and downwards double-travel mode, the anodic process took place throughout the movement time.

The machined micro-dimples’ diameter and depth were measured using a digital microscope (VHX-2000, KEYENCE, Japan) and their profile was examined by a scanning electron microscope (Merlin Compact, Carl Zeiss NTS GmbH, German). The surface roughness of the micro-dimples was measured by a white light interferometer (TALYSURF CCI6000, Taylor Hobson, UK). In order to evaluate the uniformity of geometric dimensions’ distribution of the machined micro-dimples, 10 micro-dimples were randomly selected from different positions of the workpiece and the factor CV (coefficient of variation), defined as the ratio of the standard deviation of the micro-dimple’s dimensions to their mean value, was introduced.

## 4. Results

### 4.1. Effect of the Magnetic Field

**Figure 3:**
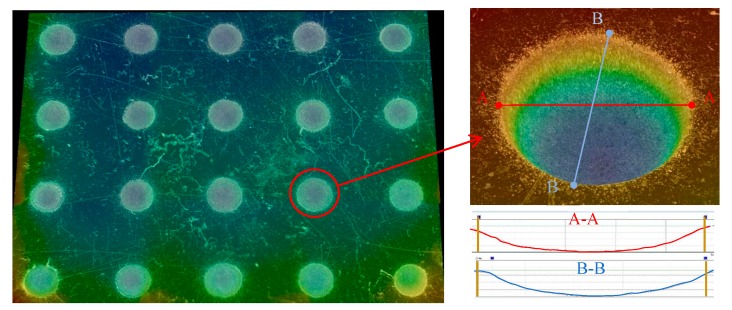
Three-dimensional profile of micro-dimple arrays machined in the single-travel mode and under the magnetic field (single-travel time of 0.3 s and 8.5 V).

**Figure 4:**
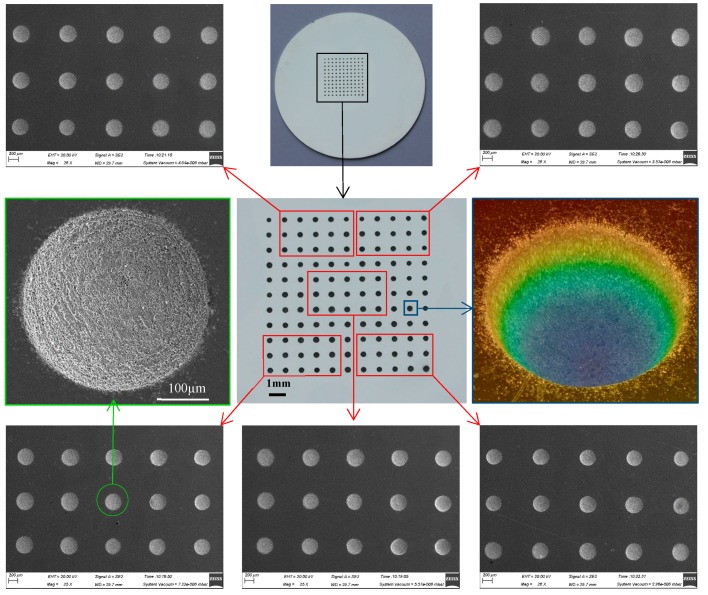
Morphologies of the arrayed micro-dimples machined in the single-travel mode and under the magnetic field (single-travel time of 0.3 s, 8.5 V).

**Figure 5:**
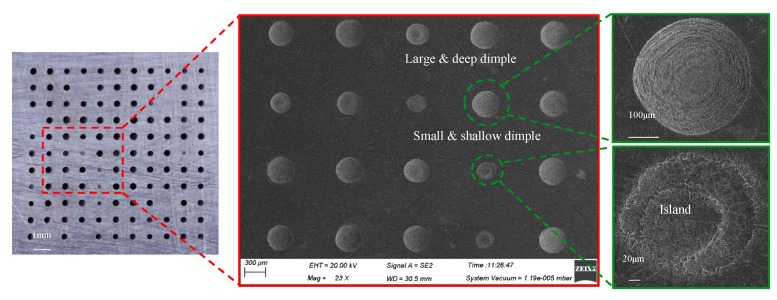
Morphologies of the arrayed micro-dimples machined in the single-travel mode and without the magnetic field (single-travel time of 0.3 s, 8.5 V).

As shown in Figure 3, the arrayed micro-dimple formed in the moving downwards single-travel mode and with the superimposition of magnetic field exhibited a highly identical three-dimensional geometric profile, showing a typical basin shape. SEM images of the micro-dimples shown in Figure 4 further demonstrate the consistency in their three-dimensional geometric profile and surface morphologies, and that their machined surfaces are smooth enough, with an Ra of 0.21–0.35 µm. In contrast, if no magnetic field was superimposed, the machined micro-dimples displayed a markedly heterogeneous distribution in their three-dimensional geometric profile and surface morphologies, as shown in Figure 5. Their surface roughness *R*a was also higher, ranging from 0.43 to 0.97 µm. 

**Figure 6:**
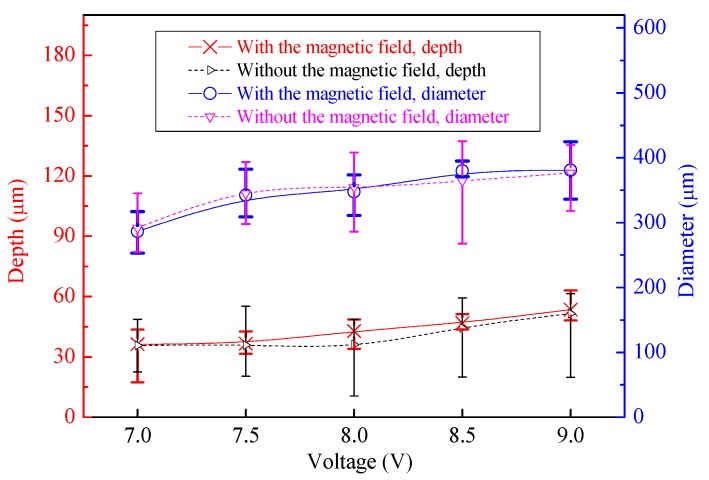
Change in dimensions of machined arrayed micro-dimples with the applied voltage and with or without magnetic field (single-travel time of 0.3 s).

**Figure 7:**
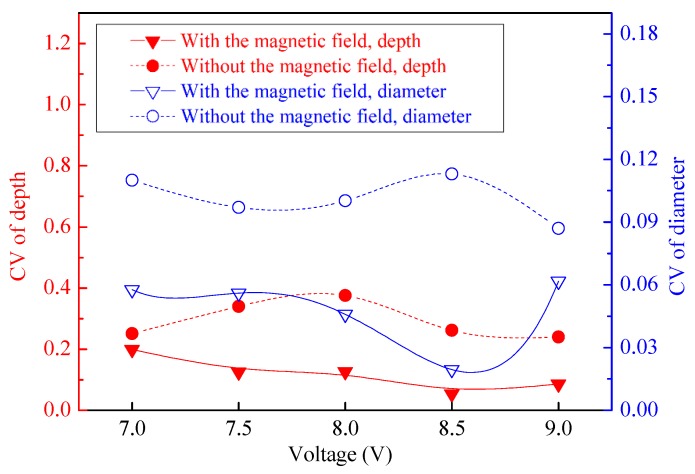
Change in dimensional CV of the arrayed micro-dimples with or without the magnetic field (single-travel time of 0.3 s).

To comparatively analyze the geometric profile distribution uniformity of the machined micro-dimples produced with or without the magnetic field, the change of the geometric dimensions with the applied voltage is plotted together in Figure 6 and Figure 7. Generally, the average dimensions including the depth and diameter increased by increasing the applied voltages. Their depths and diameters had no obvious difference at the same voltage irrespective of the presence of the magnetic field.

**Figure 8:**
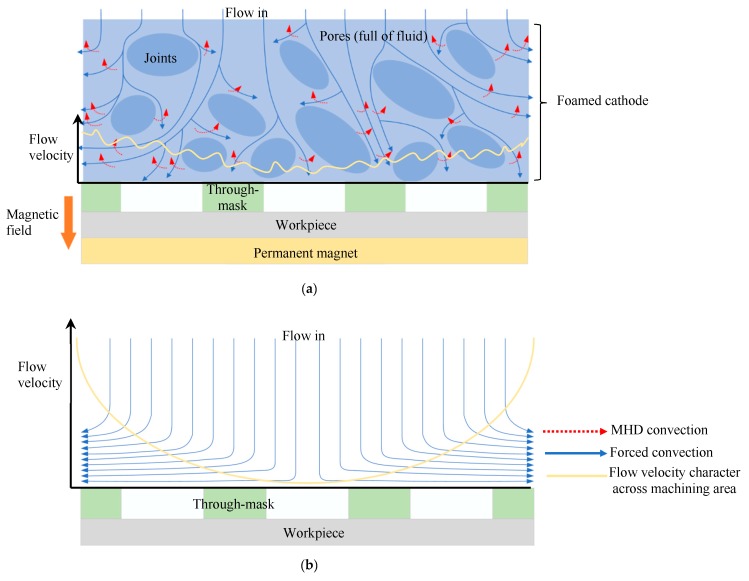
Schematic diagram of the fluid flow in the machining area (**a**) fluid flow driven by the forced convection and the magnetohydrodynamic (MHD) convection in the foamed cathode (**b**) fluid flow driven by the forced convection.

The geometric dimension CV values of the machined micro-dimples formed under the two situations differed greatly, as shown in Figure 7. The CV values of the micro-dimples obtained with the magnetic field were much smaller than those obtained without the magnetic field, which indicates that the superimposition of the magnetic field during TMEMM improves the homogeneousness of the geometric profile distribution. The positive effects on the geometric profile consistency mainly come from two aspects. On the one hand, the magnetic field force enables the through-mask to be more firmly and uniformly fixed on the workpiece. On the other hand, as shown in Figure 8, the mass transfer distribution within the interelectrode can be further homogenized with the aid of the MHD effects, which relatively raises the mass transfer efficiency of the central area in the interelectrode gap. In the conventional case without the introduction of magnetic field effects, the mass transfer differed significantly over the entire machining area in which the central area was the weakest and the margin area was the biggest, as shown in Figure 8b.

### 4.2. Effect of Machining Mode

**Figure 9:**
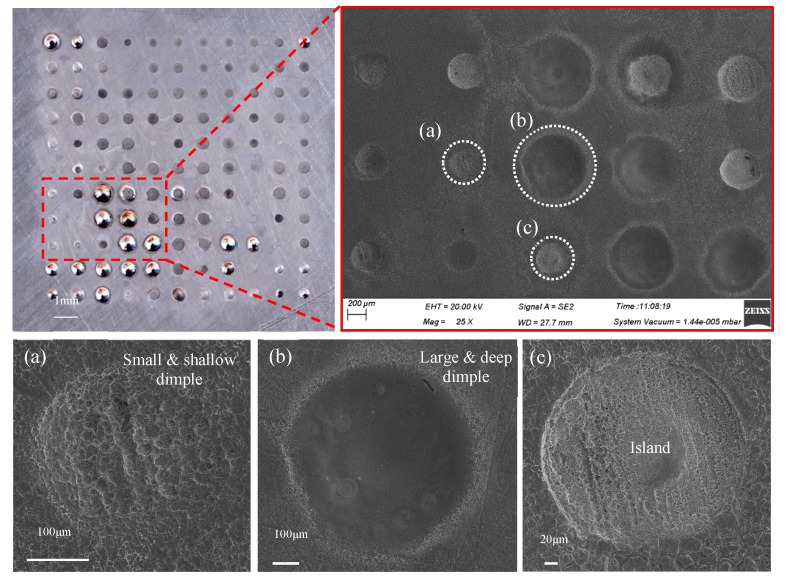
Morphologies of the arrayed micro-dimples machined in the double-travel mode and with the magnetic field (double-travel time of 0.3 × 2 s, 8.5 V).

**Figure 10:**
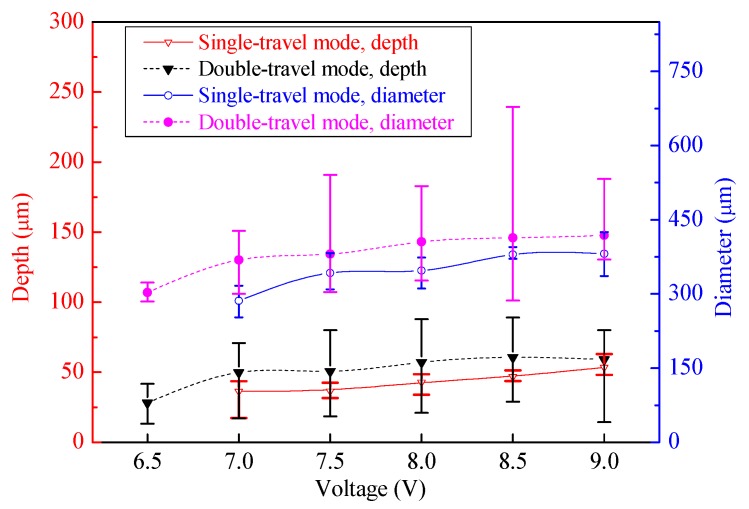
Change in dimensions of the arrayed micro-dimples machined in the single-/double-travel mode and with the magnetic field (single-travel time of 0.3 s and double-travel time of 0.3 × 2 s).

**Figure 11:**
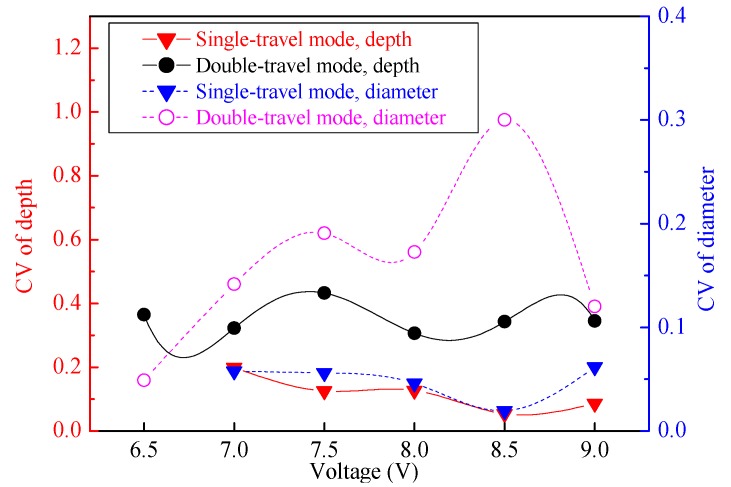
Change of dimensional coefficient of variation (CV) of the arrayed micro-dimples machined in the single-/double-travel mode and with the magnetic field (single-travel time of 0.3 s and double-travel time of 0.3 × 2 s).

**Figure 12:**
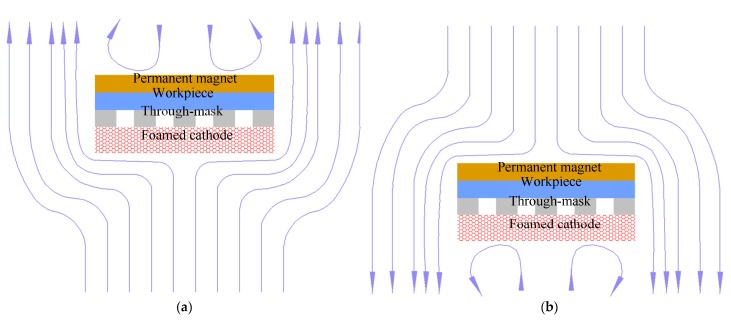
Schematic diagram of the fluid flow driven by the machining assembly moving in a linearly reciprocating way: (**a**) moving downwards; (**b**) moving upward.

**Figure 13:**
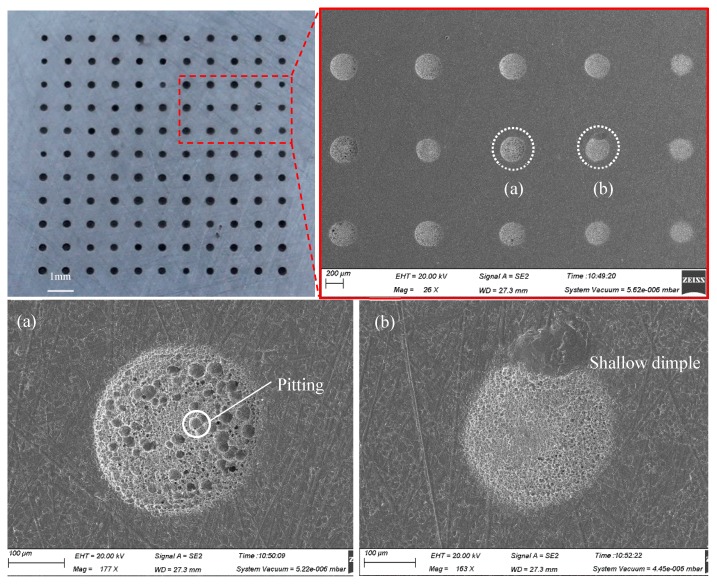
Morphologies of micro-dimple arrays machined in the double-travel mode and with the magnetic field (double-travel time of 0.3 × 2 s, 6.5 V).

As mentioned previously, two machining modes were applied to the TMEMM processes with the superimposition of the magnetic field: moving downwards single-travel and moving up-and downwards double-travel mode. Figure 9 shows the morphologies of the micro-dimples machined with the moving upwards and downwards double-travel mode. It was found that the micro-dimples showed a very nonuniform distribution in the morphology, geometric profile, and dimensions of the micro-dimples—even the magnetic field was superimposed. Particularly, islands were observed in some micro-dimples and the geometric profile and dimensions differentiated greatly. In contrast, the micro-dimples obtained with the single-travel mode were considerably homogeneous in surface morphologies and geometric profile as well as dimensions. These differences can be further verified in Figure 10 and Figure 11 in which the changes in the geometric dimensions and their CV values respective of the machined micro-dimples with the machining mode are presented. In general, the micro-dimples obtained with the single-travel machining mode were smaller and shallower and had reduced CV values compared with those obtained with the double-travel machining mode at the same voltage. The reason for these discrepancies could come from two aspects. Firstly, in the double-travel machining mode, the anodic dissolution took place in a deteriorated mass transfer environment during the moving-up travel of the machining assembly because of negative wake effects [39]. These effects often give rise to a highly ununiformly distributed flow field with a relatively slow flow velocity on the area opposite to the moving direction, as schematically depicted in Figure 12. Secondly, in the single-travel machining mode, on the contrary, there is much more time for the process to discharge the electrolytic products and to refresh the electrolyte because no current is applied to the two electrodes during the moving upward travel, and thereby the anodic dissolution always proceeds under excellent mass transfer conditions. It was further verified that, with the double-travel machining mode, even though a very small voltage (6.5 V) was applied to the TMEMM process, the machined micro-dimples were still inconsistent in their geometric profile and dimensions, as shown in Figure 13.

Therefore, for this newly proposed TMEMM process, the single-travel machining mode is recommended. In the following investigations, the single-travel machining mode was selected.

### 4.3. Effect of the Applied Voltages and the Moving Speed of the Machining Assembly

**Figure 14:**
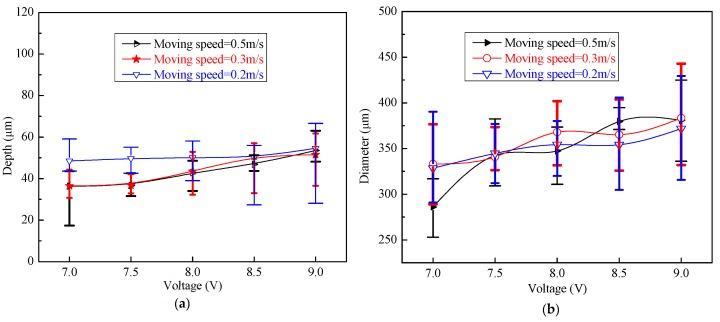
Dimensions of micro-dimples machined under different single-travel moving speeds and with the magnetic field (**a**) depth of micro-dimples (**b**) diameter of micro-dimples.

**Figure 15:**
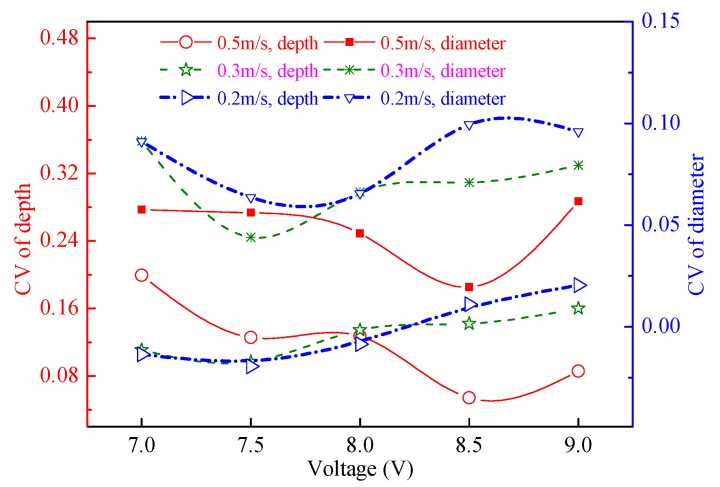
Dimensional CV of the micro-dimples machined under different single-travel moving speeds and with the magnetic field.

The dimensions of the machined micro-dimples, including their depth and diameter, increased by increasing the applied voltages, as shown in Figure 14 and Figure 15, but the growth trend of their dimensions was quite different depending on the moving speed of the machining assembly. For higher moving speeds, such as 0.5 m/s, their depth and diameter had obvious growth trends by increasing the voltages, but for low moving speeds, such as 0.2 m/s, the trends were relatively slow. This is because the slow moving speed cannot provide efficient mass transfer for the vigorous dissolution process occurring at high voltages, but if the applied voltage is appropriately small, the moving speed easily meets the requirements of the mass transfer for the dissolution reactions and the removal of the electrolytic products. In general, to make the TMEMM process accurate and cost-efficient, higher applied voltages need higher machining assembly moving speeds to match, and lower applied voltages correspond to lower moving speeds. It was further discovered from Figure 14 that the minimum CV depth and CV diameter are 5.4% and 1.9%, respectively, when the appropriate combination of the applied voltage (8.5 V) and the moving speed (0.5 m/s) are used, showing a considerably narrower variation range of 43.7–51.3 µm in depth and 370.9–394.9 µm in diameter. To the best knowledge of the authors from the reported literature, the CV values obtained in our study are sufficiently small to reach the levels that were obtained from the standard photolithographic photoresist electrochemical machining process [20,21,28], and they are also smaller than those achieved with the first version foamed cathode TMEMM process [30].

## 5. Conclusions

In this study, a modified foamed cathode through-mask electrochemical micromachining process was proposed to fabricate microstructure arrays with a higher dimensional uniformity and a better surface quality avoiding the use of the traditional pump-driven circulation system. In the modified process, the reusable mask is pressed by the magnetic field force, and the sandwich-like machining assembly moves in a linear reciprocation way to self-circulate the electrolyte within the assembly. Some experiments were carried out to verify the feasibility and applicability of this process. According to the theoretical analysis and experimental results, some conclusions are made as follows:

1. The modified foamed cathode TMEMM process facilitates the achievement of significant uniform micro-dimples with a favorable surface morphology. The coefficient of variation of the machined micro-dimple’s depth and diameter is reduced to 5.4% and 1.9%, respectively, and the minimum surface roughness *R*a is 0.21–0.35 µm. This is mainly due to the cooperation of the significantly effective fixture of the through-mask on the workpiece by the magnetic field force and the improved mass transfer driven by the MHD effects.
2. The modified foamed cathode TMEMM process is effective and practical only when it is carried out in a single-travel machining mode mainly because it eliminates the negative wake effect and the anodic dissolution process is kept in the highly efficient mass transfer state.
3. For the modified foamed cathode, the applied voltage and the moving speed of the machining assemble need be matched appropriately. That is, higher applied voltages match bigger moving speeds of the machining assembly, and lower applied voltages correspond to lower moving speeds.

## Figures and Tables

**Table 1:** Experimental conditions.

Items (Unit)	Values or Variables
Electrolyte	10 wt% NaNO_3_
Temperature (°C)	25
Material of the through-mask	Polyvinyl chloride (PVC)
Mask thickness (mm)	0.1
Mask hole diameter (mm)	0.2
Amount of mask holes	121
Magnetic flux density (T)	0.2
DC voltage (V)	6.5~9
Single-travel distance (m)	0.15
Single-travel time (s)	0.3, 0.5, and 0.7
Moving speed of machining assembly (m/s)	0.5, 0.3, and 0.2
